# 95871 Mortality in Castration-Resistant Prostate Cancer Patients with Pre-existing Cardiovascular Comorbidities Receiving Oral Androgen Signaling Inhibitors

**DOI:** 10.1017/cts.2021.719

**Published:** 2021-03-30

**Authors:** Ibrahim M. Asiri, Henry N. Young, Ronald C. Chen, Anant Mandawat, Viraj Master, Steven R.H. Beach, Ewan K. Cobran

**Affiliations:** 1 University of Georgia, Department of Clinical and Administrative Pharmacy; 2 University of Kansas, Department of Radiation Oncology; 3 Emory University, Department of Hematology and Medical Oncology; 4 Emory University, Department of Urology; 5 University of Georgia, Department of Psychology

## Abstract

ABSTRACT IMPACT: Limited research has been conducted on the survival of men with castration-resistance prostate cancer (CRPC) with a pre-existing history of cardiovascular disease, receiving oral androgen signaling inhibitors. This study highlights all-cause and prostate cancer-specific mortality for elderly patients with CRPC with pre-existing history of cardiovascular disease. OBJECTIVES/GOALS: Inadequate knowledge is known about the survival of men with castration-resistance prostate cancer (CRPC) with pre-existing history of cardiovascular disease (CVD), receiving oral androgen signaling inhibitors (OASI). We compared all-cause and prostate cancer-specific mortality for elderly patients with CRPC with pre-existing history of CVD. METHODS/STUDY POPULATION: An active comparator, new user design, was used to identify 2,608 men older than age 65 years with CRPC using the Surveillance, Epidemiology, and End Results (SEER)-Medicare linked database from 2011 to 2015. Patients were grouped into two analytical cohorts by CVD history. Within each analytical cohort patients were divided into two arms based on their new-user status (OASI vs. chemotherapy). All demographics and clinical characteristics were adjusted by inverse probability treatment weights (IPTWs). Unadjusted and IPTW-adjusted time-dependent Cox models, and Fine and Gray’s models were conducted to evaluate associations between OASI and all-cause and prostate cancer-specific mortality. RESULTS/ANTICIPATED RESULTS: Nearly 64.5% of patients had pre-existing CVD. We observed a lower all-cause mortality in the pre-existing CVD cohort compared to the no pre-existing CVD cohort (IPTW-adjusted hazard ratio [AHR], 0.59; 95% Confidence Interval [CI], 0.54 to 0.64; IPTW-AHR, 0.68; 95% CI, 0.59 to 0.78, respectively). Similarly, the prostate cancer specific-mortality was showed to be lower in the pre-existing CVD cohort compared to the no pre-existing CVD cohort when comparing OASI versus chemotherapy by the IPTW-adjusted time-dependent Fine and Gray’s models (IPTW-AHR, 0.60; 95% CI, 0.55 to 0.66; IPTW-AHR, 0.68; 95% CI, 0.59 to 0.80, respectively). DISCUSSION/SIGNIFICANCE OF FINDINGS: OASI showed a significant protective effect against all-cause and prostate cancer-specific mortality compared with chemotherapy; however, were less protective among patients without pre-existing CVD. Further studies are needed to investigate OASI in patients with and without pre-existing CVD.

